# A Nanoparticle-Coated Cellulose Acetate Membrane for Highly Efficient, Low-Cost Circulating Tumor Cell Detection

**DOI:** 10.3390/bios14100472

**Published:** 2024-10-01

**Authors:** Yize Zhao, Yaqi Pan, Hao Sun, Pengfei Huo, Guangtong Wang, Shaoqin Liu

**Affiliations:** 1 Key Laboratory of Bio-Based Materials Science & Technology (Ministry of Education), College of Materials Science and Engineering, Northeast Forestry University, Harbin 150040, China; 2022111514@nefu.edu.cn; 2 School of Life Science and Technology, Harbin Institute of Technology, Harbin 150080, China; 22s028030@stu.hit.edu.cn; 3 School of Chemistry and Chemical Engineering, Harbin Institute of Technology, Harbin 150080, China; 19b925120@stu.hit.edu.cn; 4 School of Medicine and Health, Harbin Institute of Technology, Harbin 150080, China

**Keywords:** circulating tumor cell, biosensor, nanoparticle, nanozyme, covalent organic framework

## Abstract

Detecting circulating tumor cells has exhibited great significance in treating cancers since its concentration is an index strongly associated with the development and transfer of the tumor. However, the present commercial method for CTC detection is still expensive, because special antibodies and complicated devices must be used for cell separation and imaging. Hence, it is quite necessary to apply alternative materials and methods to decrease the cost of CTC detection. In this article, we coated a cellulose acetate membrane with nanoparticles formed by the polymerization of melamine and furfural, creating a surface with nanoscale roughness for the highly efficient capture of the sparse CTCs in a blood sample. Subsequently, the CTCs on the surface can be quantitatively detected by colorimetry with the aid of a COF-based nanozyme. The detection limit (LOD) can be as low as 3 cells/mL, which is the lowest LOD among the colorimetric methods to our knowledge. Considering the low cost of fabricating the membrane for CTC capture and the robustness of nanozymes compared with natural enzymes, this CTC detection approach displays great potential to decrease the financial burden of commercial CTC detection.

## 1. Introduction

Circulating tumor cells (CTCs) are the tumor cells that have sloughed off the primary tumor to extravasate into and circulate in the blood [1,2,3]. They are an important index that indicates the development and transfer of the tumor. However, the detection of CTCs remains challenging, because even in the blood of most cancer patients the concentration of CTCs is far less than 10 cells/mL. The present commercial method for CTC detection involves special antibodies and complicated devices for cell separation and imaging; hence, the price of CTC detection is still high. For example, a single examination usually costs at least RMB 3000 in China. However, the concentration of CTCs needs to be continually measured to monitor the therapeutic effect; thus, it will bring a significant financial burden to the patients. To decrease the cost, many alternative approaches to detecting CTCs have been developed [4,5,6].

Since CTCs are rare in blood and their concentration is far less than that of hemocytes, enrichment is highly requisite for detecting CTCs. Antibody-modified magnetic beads are usually applied to collect CTCs from blood samples [7,8]. However, the use of fragile, expensive antibodies significantly increases the price of the detection. Therefore, it is still crucial to develop alternative methods for enriching CTCs. S. Wang et al. reported that a surface with micro- or nano-scaled roughness is able to capture many more cells than a flat surface [9]. Cutting-edge techniques including electrodeposition, photolithography, etching, and nanoimprinting have been applied to fabricate such a surface for cell enrichment [10]. However, all of these methods exhibited non-ignorable disadvantages. Photolithography and nanoimprinting require special instruments and are difficult to mass produce. Electrodeposition usually needs noble metals, and the resulting micro- or nano-scaled protrusions are generally quite fragile. Hazardous chemicals are inevitable in the etching method. Hence, exploring facile and low-cost approaches to produce a surface with micro- or nano-scaled roughness is still necessary.

The enriched CTCs can be quantitatively detected by various methods including cell counting [11,12], colorimetry [13,14,15], electrochemistry [10,16,17,18,19], etc., among which the colorimetric method is the easiest and most reliable. It is usually realized by an enzyme-labeled probe that can specifically recognize the tumor cells. Meanwhile, the enzyme, for example horseradish peroxidase, can catalyze a chemical reaction between H_2_O_2_ and 3,3′,5,5′-Tetramethylbenzidine (TMB), yielding a colored oxidation product (oxTMB). In recent years, nanozymes, which are a good low-cost alternative to natural enzymes, have been widely reported [20,21,22]. They exhibit excellent catalytic capacity comparable to natural enzymes but are much more robust and easier to preserve. Various nanomaterials, such as particles [23], MOFs [24,25,26], COFs [27,28], etc., have been developed as nanozymes. For example, H. Wei reported an MOF-based glutathione peroxidase-mimicking metal organic framework nanozyme, and the activity of such nanozyme can be tuned by substituted ligands [25]. A nanozyme with an -NH_2_ ligand showed a potential application in anti-inflammation therapy. Covalent organic frameworks (COFs) have also been used for the fabrication of nanozymes. In 2020, X. Qu demonstrated a chiral COF-based nanozyme with ultrahigh horseradish peroxidase-mimicking activity. It possessed 21.7 times higher activity than natural HRP [27].

**Figure 1:**
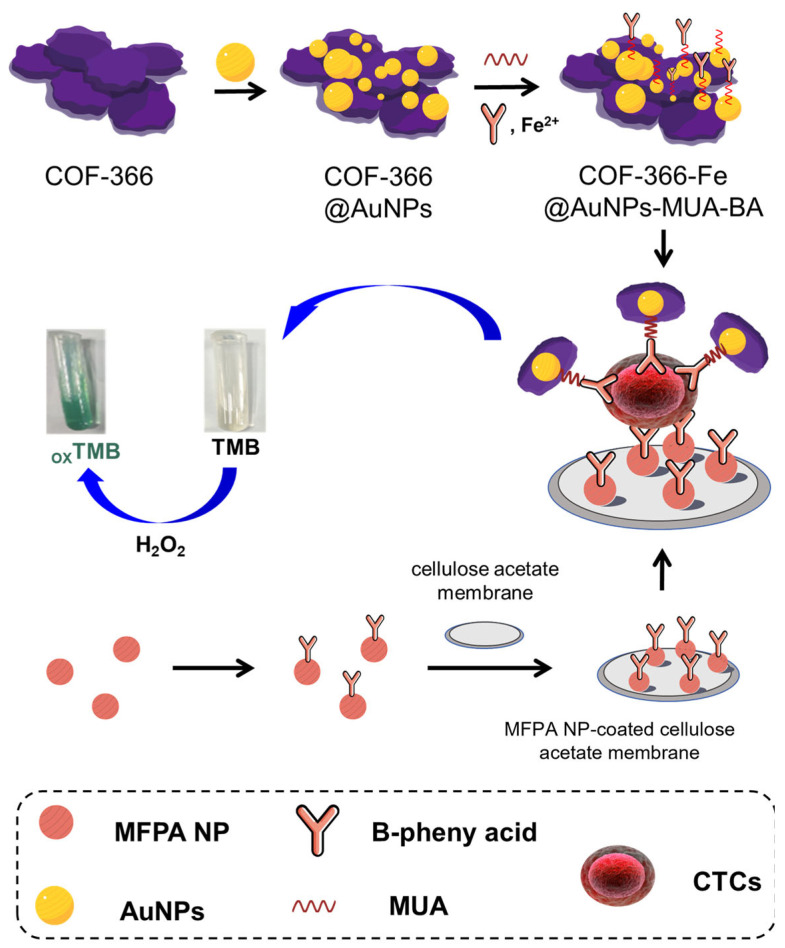
A schematic illustration of the low-cost, easily operated, and reliable CTC detection platform.

Herein, we report a low-cost platform for the fast detection of CTCs. (Figure 1) The CTCs in blood are first captured on the surface of a cellulose acetate membrane, which is coated with phenylboronic acid-modified nanoparticles formed by the polymerization of melamine and furfural (MFPA NPs) [28]. The MFPA NPs on the surface create a huge number of nanoscale protrusions on the surface of the membrane. The experimental results exhibited that the increase in the surface roughness can significantly benefit the adsorption of the cancer cell. The phenylboronic acid on the particles can specifically and tightly immobilize the captured cancer cells in blood due to the excessive sialic acid on their cytomembranes [29], while the hemocytes deposited on the membrane are easily washed off. The CTCs adsorbed on the membrane can be probed by a phenylboronic acid-modified COF-based nanozyme which can catalyze the oxidation of TMB to oxTMB. oxTMB can be quantitatively monitored to determine the number of cancer cells. The platform we reported shows the following advantages: (a) The use of an MFPA NP-coated cellulose acetate membrane and a COF-based nanozyme remarkably decreases the cost. MFPA NPs can be mass-produced from low-priced compounds by one-pot synthesis. The MFPA NP-coated membrane was simply prepared by vacuum filtration. It is much more convenient than other methods for creating nanoscale roughness on flat surfaces, such as photolithography, etching, electrodeposition, and nanoimprinting. The preservation cost of COF-based nanozymes is much less than similar detection agents containing an antibody or enzyme, which usually need careful cryopreservation. (b) The quantity of CTCs is determined by colorimetry, which is easier to operate and more reliable than electrochemical methods such as voltammetry and electrochemical impedance spectroscopy. Meanwhile, compared with cell counting and inductively coupled plasma mass spectrometry (ICP-MS), the instrument for colorimetry is inexpensive and easy to maintain. (c) The phenylboronic acid groups on the MFPA NPs and COF-based nanozyme can specifically recognize the cancer cells, avoiding the interference of the hemocytes. Hence, the approach reported in this article shows great potential to remarkably reduce the cost of commercial CTC detection.

## 2. Materials and Methods

### 2.1. The Preparation of COF-366 

**Figure 2:**
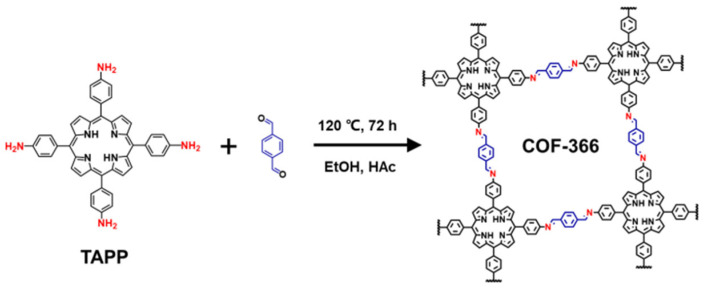
The synthesis of COF-366.

COF-366 was synthesized according to the previous literature with a slight change [30]. As shown in Figure 2, 27.8 mg of 4-(10,15,20-Triphenyl-21H,23H-porphin-5-yl)benzenamine (TAPP) and 10.8 mg of terephthalaldehyde were added in a Teflon-lined autoclave. Then, 1 mL of mesitylene, 1 mL of anhydrous ethanol, and 0.2 mL of acetic acid were added as solvents. The reaction mixture was degassed and heated at 120 °C for 72 h under sealed conditions. The product was washed with 1,4-dioxane, tetrahydrofuran, and acetone, and finally vacuum-dried at 60 °C for 12 h, yielding purified COF-366, a dark purple powder.

### 2.2. The Preparation of COF-366@AuNPs

A total of 10 mg of COF-366 was dispersed in 10 mL of ddH_2_O, and 1.2 mL of 0.6 mM sodium citrate was added. The mixture was heated to boiling. Next, 0.6 mL of 1% (wt %) chloroauric acid was added immediately and the mixture was continuously heated for 15 min. The product was collected by centrifugation and washed with deionized water. Finally, COF-366@AuNPs were dried by vacuum at 60 °C for 12 h.

### 2.3. The Preparation of COF-366@AuNPs-MUA

A total of 10 mg of COF-366@AuNPs was dispersed in 10 mL of ultrapure water. Then, 20 mg of 11-mercaptoundecanoic acid (MUA) dissolved in 10 mL of anhydrous ethanol and 10 mL of 16 μM NaOH were subsequently added. The mixture was heated to 75 °C and stirred for 1.5 h. The precipitate was collected and washed three times with anhydrous ethanol. The product was finally vacuum-dried at 40 °C for 12 h.

### 2.4. The Preparation of COF-366@AuNPs-MUA-BA

A total of 10 mg of COF-366@AuNPs-MUA was first dispersed in 10 mL of ultrapure water. Then, 5 mL of 5 mM 1-(3-dimethylaminopropyl)-3-ethylcarbodiimide and 5 mL of 5 mM N-hydroxy succinimide solution were added. After 1 h of stirring, 17 mg of aminophenyl boronic acid (BA) dissolved in a 10 mL mixture of CH_3_OH and H_2_O (v:v = 3:7) was added. The mixture was continuously stirred at room temperature for 24 h. The product was collected by centrifugation and washed with dichloromethane and methanol. The product was finally vacuum-dried at 40 °C for 12 h.

### 2.5. The Preparation of COF-366-Fe@AuNPs-MUA-BA

A total of 10 mg of COF-366@AuNPs-MUA-BA was dispersed in 10 mL of ultrapure water. Next, 13 mg of FeSO_4_·7H_2_O dissolved in a 12 mL mixture of dichloromethane and methanol (v:v = 7:3) was added. The mixture was stirred at room temperature for 36 h to obtain the final product COF-366-Fe@AuNPs-MUA-BA.

### 2.6. Coating the Cellulose Acetate Membrane with Melamine–Furfural Phenylboronic Acid (MFPA) Particles

**Figure 3:**
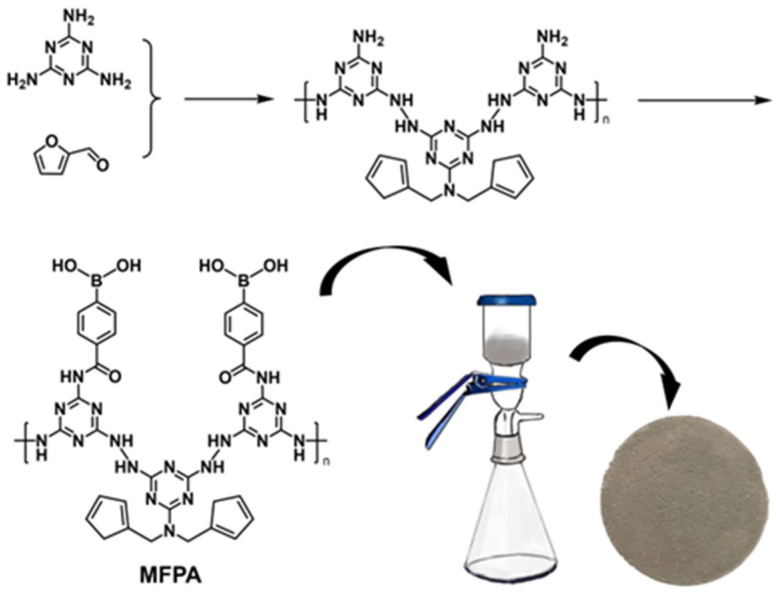
Coating a cellulose acetate membrane with melamine–furfural phenylboronic acid (MFPA) particles.

The synthesis route of MFPA is shown in Figure 3. A total of 0.786 g of melamine was added to 32 mL of ddH_2_O and stirred at 80 °C for at least 10 min to make the melamine completely dissolve. Next, 0.6 g of furfural was subsequently added and the mixture was transferred to a pressure kettle and heated at 180 °C for 16 h. The yielded solid was collected by filtration and redispersed in 20 mL of ddH_2_O. Next, 19 mg of *o*-carboxyphenylboronic acid dissolved in a 10 mL mixture of dichloromethane and methanol (v:v = 7:3) and 5 mL of aqueous solution containing 5 mM EDC and 5 mM NHS were premixed to activate the carboxy groups. After 1 h, the above mixture was added to the dispersion of the solid product obtained by the hydrothermal reaction. After 4 h of stirring at room temperature, MFPA particles were obtained and separated by centrifugation. The MFPA particles were deposited on the cellulose acetate membrane by suction filtration. Next, 15 mL of the dispersion with a certain amount of MFPA particles was filtrated by the cellulose acetate membrane, yielding the MFPA-coated membrane shown in Figure 3. 

### 2.7. Cell Culture

The MCF-7 cell line was provided by Pricella, Ltd., Wuhan, China. Cell culture was performed in a 25 cm^2^ disposable plastic culture flask in a CO_2_ incubator at 37 °C with 5% CO_2_. The cell morphology was observed using an inverted phase-contrast microscope. The color of the culture medium was observed every day. The culture medium was changed when its red color faded to yellow. 

### 2.8. Enrichment and Detection of CTCs

The performance of the CTC detection platform was verified by detecting a known number of MCF-7 cells in phosphate buffer (PBS) and rabbit blood. The suspensions of MCF-7 cells were prepared after 10 min of trypsin digestion. The concentration of the cells was counted with a cell count chamber. A series of cell suspensions with different concentrations was prepared. Then, 0.3 mL of the above suspensions was added to 9.7 mL of PBS or rabbit blood. 

For CTC detection, 60 µg of COF-366-Fe@AuNPs-MUA-BA (suspended in 1 mL of PBS) was added and incubated with the sample containing MCF-7 cells for 1.5 h. The mixture was transferred to a centrifuge tube in which an MFPA-coated cellulose acetate membrane (a circle with a diameter of 1 cm) was located in the bottom. The mixture in the centrifuge tube was sonicated for 3 min, then centrifuged at 1200 rpm for 4 min. The supernatant was removed. Subsequently, the remaining product in the tube was washed with 3 mL of ddH_2_O twice or thrice to remove the hemocytes and redundant COF-366-Fe@AuNPs-MUA-BA on the membrane. The membranes were collected and transferred to a 6-well plate. Next, 2 mL of 2 mM TMB, 2 mL of 200 μM H_2_O_2_, and 0.3 mL of acetic acid were added. After 30 min, 0.2 μL of liquid was taken to a 96-well tissue culture plate to test the absorbance at 400–800 nm with a microplate reader.

### 2.9. Adsorption of Hemocytes on MFPA NP-Coated Cellulose Acetate Membrane

To investigate the adsorption and removal of hemocytes in rabbit blood on the MFPA NP-coated cellulose acetate membrane, 0.3 mL of PBS was added to 9.7 mL of rabbit blood. The rabbit blood was pre-stained with 0.6 mL of 5 μM DiD at 37 °C for 1 h. Then, 60 μg of COF-366-Fe@AuNPs-MUA-BA (suspended in 1 mL PBS) was added. The mixture was transferred into a centrifuge tube in which an MFPA-coated cellulose acetate membrane (a circle with a diameter of 1 cm) was located in the bottom. The mixture in the centrifuge tube was sonicated for 3 min, then centrifuged at 1200 rpm for 4 min. The supernatant was removed. Subsequently, the remaining product in the tube was washed with 3 mL of ddH_2_O twice or thrice. The membrane was observed by a laser confocal microscope to verify the removal of the hemocytes on the membrane.

## 3. Results and Discussion

### 3.1. The Characterization of COF-366

**Figure 4:**
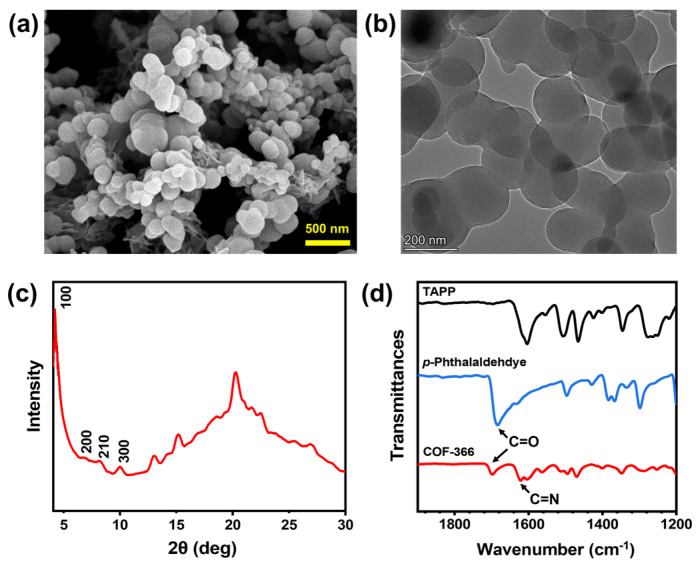
(**a**) SEM image of COF−366 particles. (**b**) TEM image of COF−366 particles. (**c**) XRD characterization of COF−366. (**d**) FTIR spectra of TAPP, *p*-phthalaldehdye, and COF−366.

The obtained COF-366 was firstly observed by TEM and SEM. As shown in Figure 4a,b, regular disk-like particles with a diameter of about 200 nm can be found. This indicates the formation of a COF since COF-366 is a highly crystallized material. The crystallinity of COF-366 was subsequently characterized by powder X-ray diffraction (PXRD). As shown in Figure 4a, the peaks at 4.2°, 6.8°, 8.2°, and 10.0° can be found, indicating its good crystalline structure. They represent the (100), (200), (210), and (300) crystal faces of COF-366, respectively [30]. The formation of COF-366 can also be verified by Fourier transform infrared spectroscopy (FTIR). As shown in the FTIR spectra in Figure 4b, compared with the monomers, a new absorption band at 1610 cm^−1^ can be found in the FTIR spectrum of COF-366, indicating the formation of the imine bond in the COF skeleton. 

### 3.2. The Characterization of COF-366-Fe@AuNPs-MUA-BA

**Figure 5:**
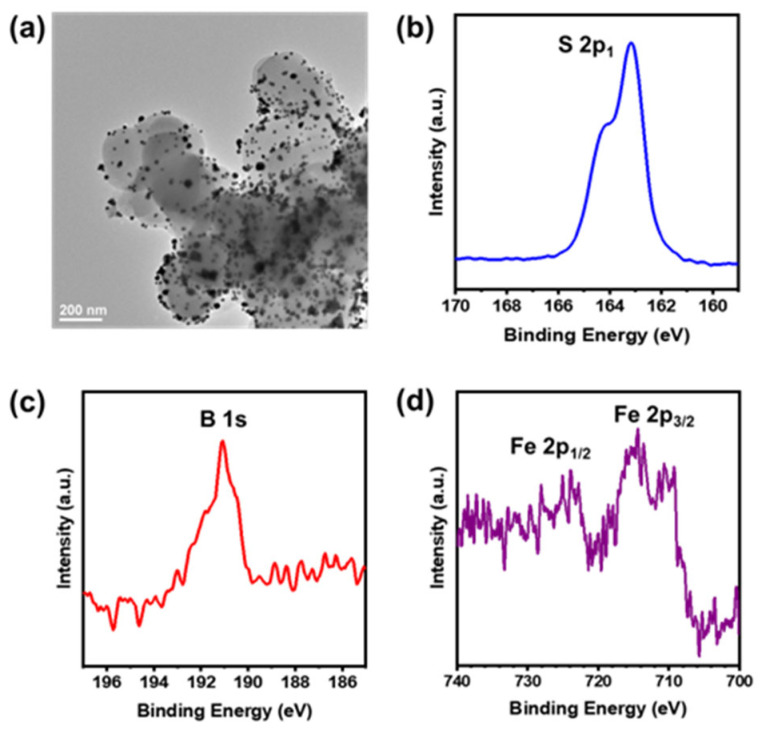
(**a**) TEM images of COF-366@AuNPs. (**b**) Characterization of S on COF-366-Fe@AuNPs-MUA-BA using XPS. (**c**) Characterization of B on COF-366-Fe@AuNPs-MUA-BA using XPS. (**d**) Characterization of Fe on COF-366-Fe@AuNPs-MUA-BA using XPS.

The Au nanoparticles (AuNPs) grown in COF-366 are shown in Figure 5a. It can be observed in the TEM image that AuNPs are located on the surface of the COF-366 nanoparticles. Subsequently, the AuNPs were modified with MUA and BA. The MUA modification can be characterized by FTIR. As shown in Figure S1, the peak of -COOH at around 3400 cm^−1^ and the peaks of C-H at 2916 cm^−1^ and 2848 cm^−1^ indicate the successful immobilization of MUA. Meanwhile, the X-ray photoelectron spectroscopy (XPS) spectrum (Figure 5b) displays a significant peak of S, further confirming the binding of MUA on AuNPs. The XPS peak of B can be found after the modification with BA (Figure 5c). Finally, the XPS spectra of COF-366-Fe@AuNPs-MUA-BA (Figure S2 and Figure 5d) exhibit the peaks of Fe 2p_3/2_ and Fe 2p_1/2_. This indicates the adsorption of Fe ions on the porphyrin units of COF-366, as well as the existence of both Fe (II) and Fe (III), which results in the good catalytic capacity of the redox reaction between H_2_O_2_ and TMB. COF-366-Fe@AuNPs-MUA-BA was also characterized by X-ray energy dispersive spectroscopy (EDS) analysis with SEM. All of the elements that compose COF-366-Fe@AuNPs-MUA-BA can be found (Figure S3).

### 3.3. The Characterization and Optimization of the MFPA NP-Coated Cellulose Acetate Membrane

**Figure 6:**
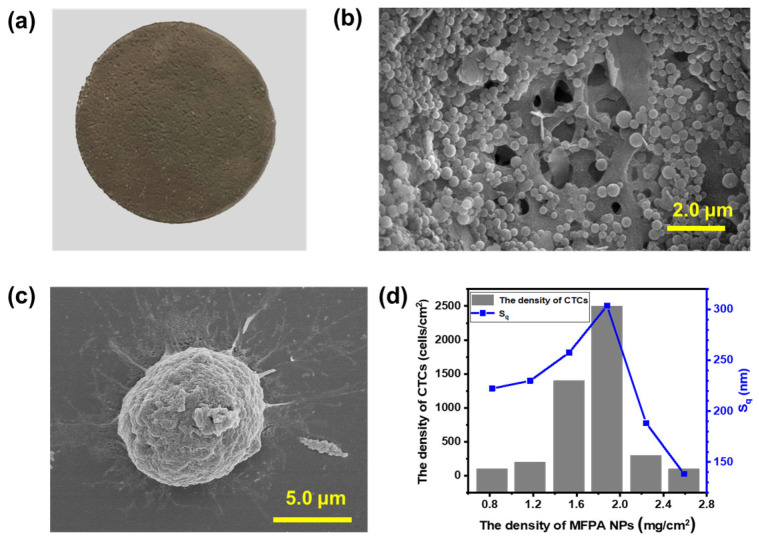
(**a**) A photograph of the MFPA NP-coated cellulose acetate membrane. (**b**) The morphology of the MFPA NPs observed by SEM. (**c**) The SEM image of the MCF-7 cells on the surface of the membrane. (**d**) The surface roughness obtained by AFM, where the cell adsorption changed with the amount of MFPA NPs deposited on the membrane surface.

The MFPA NP-coated cellulose acetate membrane is exhibited in Figure 6a. The morphology and size of the MFPA NPs on the membrane are shown in Figure 6b and Figure S4. MFPA formed perfect spherical NPs with a diameter of about 300 nm. The particles are tightly deposited on the cellulose acetate membrane, creating a rough surface with nanoscale protrusions that can facilitate the adhesion of cancer cells. The phenylboronic acid group on the MFPA NPs can covalently bind with the sialic acid overexpressed on the cytomembranes of the MCF-7 cells, while the hemocytes can only adsorb on the membrane weakly. As shown in Figure S5, the hemocytes deposited on the membrane can be easily washed off. The SEM image of the MCF-7 cells on the rough surface is shown in Figure 6c. Extended pseudopodia that bind on the protrusions can be observed. Furthermore, we found that cell adsorption changed with the amount of MFPA NPs deposited on the membrane because it determines the roughness. Atomic force microscope (AFM) characterization exhibited that the increase in the amount of MFPA NPs on the membrane results in the enhancement of roughness compared to when the MFPA NPs are few (Figure 6d). However, superfluous MFPA NPs will lead to an even and flat coating that can still be unbeneficial to the adsorption of the cancer cells (Figure 6d, Figures S6 and S7). Therefore, as shown in Figure 6d, both the surface roughness of the membrane and the number of the adhered cells reach a maximum when the MFPA NP sediment is 1.9 mg cm^−2^.

### 3.4. The Detection of CTCs

**Figure 7:**
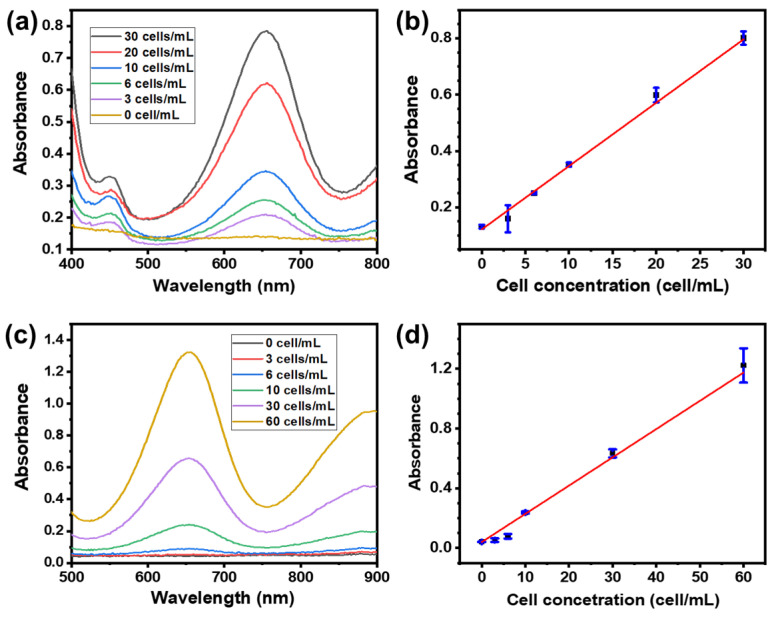
(**a**) UV spectra of the sample oxTMB generated by using different numbers of CTC cells in PBS. (**b**) The linear relationship between cell concentration in PBS and the UV absorbance at 652 nm. (**c**) UV spectra of the sample oxTMB generated by using different numbers of CTC cells in rabbit blood. (**d**) The linear relationship between cell concentration in rabbit blood and the UV absorbance at 652 nm.

The concentration of CTCs in blood is less than 10 cells/mL; hence, CTCs should be enriched before being detected. As shown in Figure 7a, we used the cellulose acetate membrane coated with 1.9 mg cm^−2^ of MFPA NPs to absorb the rare CTCs from the PBS or rabbit blood. Then, COF-366-Fe@AuNPs-MUA-BA was used as a probe to mark the CTCs captured on the membrane. COF-366-Fe, as a nanozyme, can catalyze the oxidation of TMB. This means that the quantity of CTCs can be measured by the optical absorbance of oxTMB. Figure 7 and Figure S5 display the enhancement of the UV-Vis absorbance with the increasing concentration of MCF-7 cells in the PBS or rabbit blood. An excellent linearity can be found in the range of 3–30 cells/mL. In the PBS sample, the linear equation is Abs = 0.0225C_cell_ + 0.122 (r^2^ = 0.994), while in the rabbit blood sample, the linear equation is Abs = 0.0189C_cell_ + 0.040 (r^2^ = 0.958). The LOD can be as low as 3 cells/mL. As shown in Table 1, to our knowledge, compared with the other literature on CTC sensors using the colorimetric method, this work displayed the lowest LOD. Meanwhile, compared with the previously reported electrochemical approaches, our method still exhibited a high sensitivity. In addition, the instrument for the colorimetric method is much more inexpensive and robust than that for cell counting and the IPC-MS method. Considering the cheap materials and instrument and the simple operation, the platform we herein reported is a promising alternative option for realizing low-cost CTC detection in blood.

## 4. Conclusions

In summary, we fabricated an MFPA NP-coated cellulose acetate membrane for effectively enriching CTCs from blood. We found that depositing a moderate amount of MFPA NPs on the membrane can significantly enhance its surface roughness, greatly facilitating the enrichment of cancer cells. The captured CTCs on the membrane can be detected by colorimetry using a nanozyme based on COF-366 as a probe. The linear range is 3–30 cells/mL and the LOD can be as low as 3 cells/mL. Notably, unlike rough surfaces for capturing cancer cells achieved by other techniques, the preparation of the MFPA NP-coated membrane is extremely low-cost. The MFPA nanoparticles can be massively synthesized from chemicals that can be inexpensively produced by modern industry, and the coating is easily achieved by vacuum filtration. The COF-based nanozyme probe is much easier to preserve than those containing natural enzymes. Meanwhile, it can be expected that its synthesis will be further optimized to decrease the cost and save time in large-scale production. Furthermore, the method we report in this article does not include complicated or time-consuming operations. Hence, it shows good potential as a low-cost alternative to the present commercial CTC detection methods.

## Figures and Tables

**Table 1:** Recently reported biosensors for detecting CTCs.

Material for Enrichment	Detection Method	Nanomaterial for Generating Signal	LOD (Cells/mL)	Linear Range (Cells/mL)	Ref.
Magnetic bead	Electrochemistry	AuIrPt nanozymes	5	5–10^6^	[16]
CeMOF-Au/Ketjen Black	Electrochemistry	PdPtCuRu MNSs	10	10–2 × 10^4^	[17]
Au NP/α-Fe_2_O_3_ dendritic aptamer–DNA concatemer	Photoelectrochemistry	CdSe@CdS QDs–Ab	3	300–6 × 10^5^	[31]
SMB-Apt1/S5, SMB-Apt2/S6	Electrochemistry	Au@COF-ZU1@Ru	2	8–1 × 10^5^	[18]
Immobilized antibody	Electrochemistry	polycrystalline Cu^2+^-NMOFs	5	20–10^4^	[19]
Magnetic bead	Electrochemistry/colorimetry	AgNPs/CuO NPs	5	5–10^5^	[13]
Bifunctional aptamer	Electrochemistry	Single-stranded aptamer	4	10–10^3^	[32]
Magnetic bead	Electrochemistry/colorimetry	Fe-MOF-PBA	3	10–10^5^	[15]
Bio-EpCAM aptamer	Colorimetry	MOF@Pt@MOF-H2	5	5–5 × 10^5^	[33]
AuNP aptamer nanoconjugates	Colorimetry	TP/SYL3C-MoS_2_ or CUR/C-9S-MoS_2_ NFs	5	5–5 × 10^4^	[34]
Fe_3_O_4_-SiO_2_-Gel/P1/mDNA	Colorimetry	SWCNT colorimetric probe	10	10–500	[35]
mucin 1 aptamer	Fluorescence	CdTe QDs	3	10–10^5^	[36]
Stepwise centrifugation	ICP-MS	CuS NPs, C-Ag+ -C; Hg^2+^, aptamer; CHA	1	1–100	[37]
Magnetic bead-Apt-FAM-Au NP	ICP-MS	Au NP-modified aptamer	81	2 × 10^2^–1.2 × 10^4^	[38]
Magnetic-bead-based dual aptamers	ICP-MS	Dual-metal nanoparticles	50	100–6 × 10^3^	[39]
MFPA NP-coated cellulose acetate membrane	Colorimetry	COF-366-Fe@AuNPs-MUA-BA	3	3–30	This work

## Data Availability

The data are contained within the article or Supplementary Material. The original contributions presented in this study are included in the article/Supplementary Materials; further inquiries can be directed to the corresponding authors.

## Supplementary Materials

The following supporting information can be downloaded at https://www.mdpi.com/article/10.3390/bios14100472/s1: Figure S1: FTIR spectra of COF-366-Fe@AuNPs-MUA, MUA, and COF-366; Figure S2: XPS spectra (survey) of COF-366-Fe@AuNPs-MUA-BA; Figure S3: SEM-EDS images of COF-366-Fe@AuNPs-MUA-BA; Figure S4: Gaussian distribution of diameter of MFPA NPs; Figure S5: The laser confocal microscope images of the MFPA NP-coated cellulose acetate membrane after removing hemocytes by washing; Figure S6: The AFM images of the cellulose acetate membrane coated by different amounts of MFPA NPs; Figure S7: The fluorescent microscope images of the MCF-7 cells adsorbed on the MFPA NP-coated membrane with different amounts of MFPA; Figure S8: The enhancement of the UV-Vis absorbance with the increasing concentration of MCF-7 cells in PBS or rabbit blood.
