# Supplementary material for: A Nanoparticle-Coated Cellulose Acetate Membrane for Highly Efficient, Low-Cost Circulating Tumor Cell Detection
Source: Biosensors (Basel). 2024 Oct 1;14(10):472. doi: 10.3390/bios14100472 (PMC11505997; doi:10.3390/bios14100472)
Supplement: Supplementary file 1 [file biosensors-14-00472-s001.zip › biosensors-3184011-supplementary.pdf]

biosensors

MDPI

Article

A Nanoparticle-Coated Cellulose Acetate Membrane for Highly Efficient, Low-Cost Circulating Tumor Cell Detection

Yize Zhao [1], Yaqi Pan [2], Hao Sun [3], Pengfei Huo [1,*], Guangtong Wang [4,*] and Shaoqin Liu [4,*]

[1] Key Laboratory of Bio-based Materials Science & Technology (Ministry of Education), College of Materials Science and Engineering, Northeast Forestry University, Harbin 150040, China; 2022111514@nefu.edu.cn
[2] School of Life Science and Technology, Harbin Institute of Technology, Harbin 150080, China; 22s028030@stu.hit.edu.cn
[3] School of Chemistry and Chemical Engineering, Harbin Institute of Technology, Harbin 150080, China; 19b925120@stu.hit.edu.cn
[4] School of Medicine and Health, Harbin Institute of Technology, Harbin 150080, China
* Correspondence: huopengfei@nefu.edu.cn (P.H.); wgt@hit.edu.cn (G.W.); shaoqinliu@hit.edu.cn (S.L.)

1. Material and Instrument

4-[10,15,20-tris(4-aminophenyl)-21,24-dihydroporphyrin-5-yl]aniline, 11-mercaptoundecanoic acid, methanol, 1-(3-Dimethylaminopropyl)-3-ethylcarbodiimide hydrochloride (EDC) and N-hydroxysuccinimide (NHS) were purchased from Macklin. p-Phthalaldehyde, 1,4-dioxane, mesitylene, sodium citrate, iron(II) sulfate heptahydrate, dichloromethane, and melamine were purchased from Aladdin. Acetone, ethanol, tetrahydrofuran, and acetic acid were purchased from Fuyu Chemical (Tianjin, China). Tetrachloroauric acid was purchased from Leyan Chemical. 3-Aminobenzeneboronic acid and sodium hydroxide were purchased from Rhawn. Furfural was purchased from Innochem.

TEM image was obtained by a FEl Tecnai G2 F30 of Transmission Electron Microscope. FTIR spectra were obtained by Nicolet iN10 of Fourier Transform Infrared Spectrometer. CLSM observation was conducted by a Leica TCS SPE of Confocal Laser Scanning Microscope. The UV-Vis absorbance was measured by Tecan Spark of Microplate Reader. SEM image was obtained by a JSM-7500F Scanning Electron Micrometer. The fluorescence spectra were ThermoFisher of Fluorescence Micrometer. XRD spectra were obtained by a Bruker D8 Advance X-ray Powder diffractometer. XPS spectra were Thermo escalab 250XI of X-ray Photoelectron Spectrometer. Sonication treatment was performed by an AS2060B ultrasonic cleaner of Tianjin Automatic Science Instrument Co., ltd. Ultrasound power is 40 kHz, 30 W.

2. FTIR spectra of COF-366-Fe@AuNPs-MUA

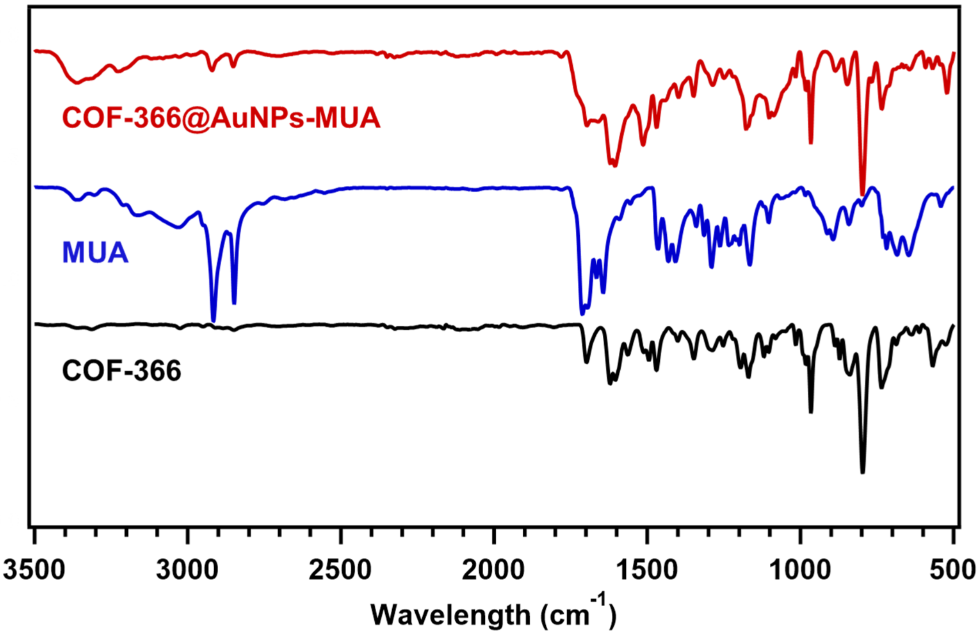

Figure S1. FTIR spectra of COF-366-Fe@AuNPs-MUA, MUA, and COF-366.

3. XPS spectra of COF-366-Fe@AuNPs-MUA-BA

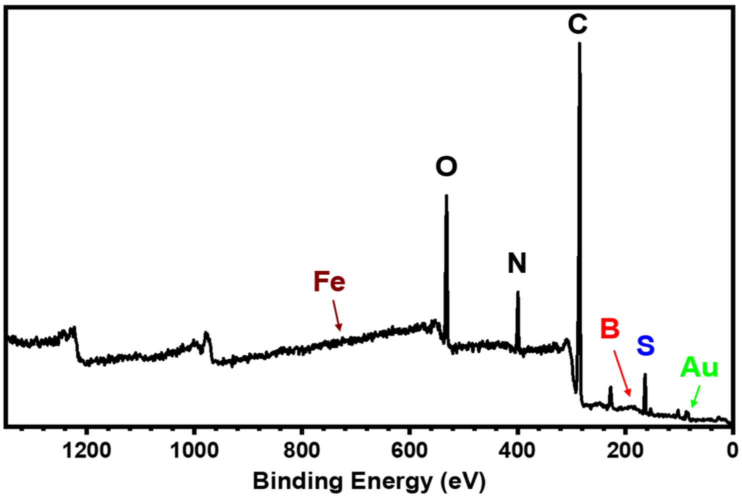

Figure S2. XPS spectra (survey) of COF-366-Fe@AuNPs-MUA-BA.

4. SEM-EDS images of COF-366-Fe@AuNPs-MUA-BA

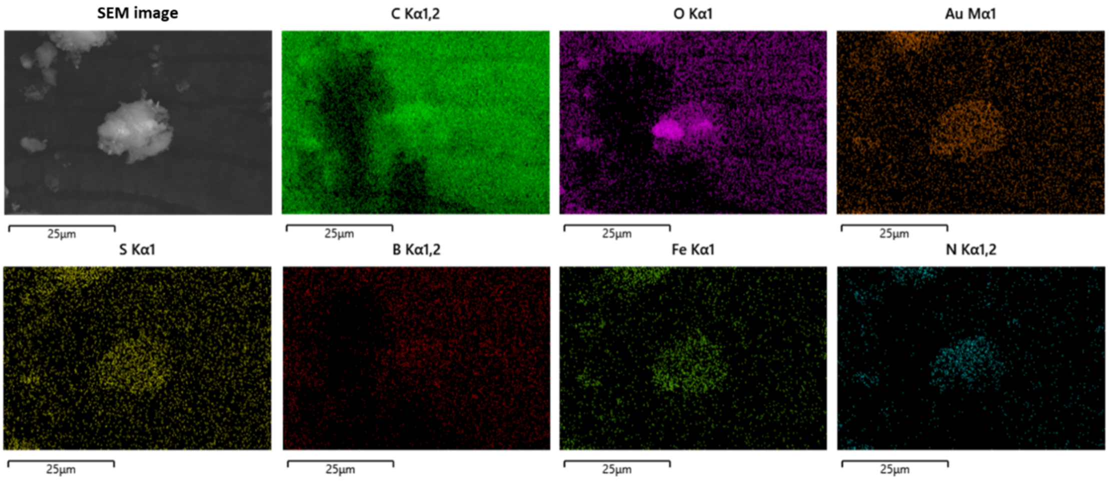

Figure S3. SEM-EDS images of COF-366-Fe@AuNPs-MUA-BA.

5. Size distribution of the MFPA NPs

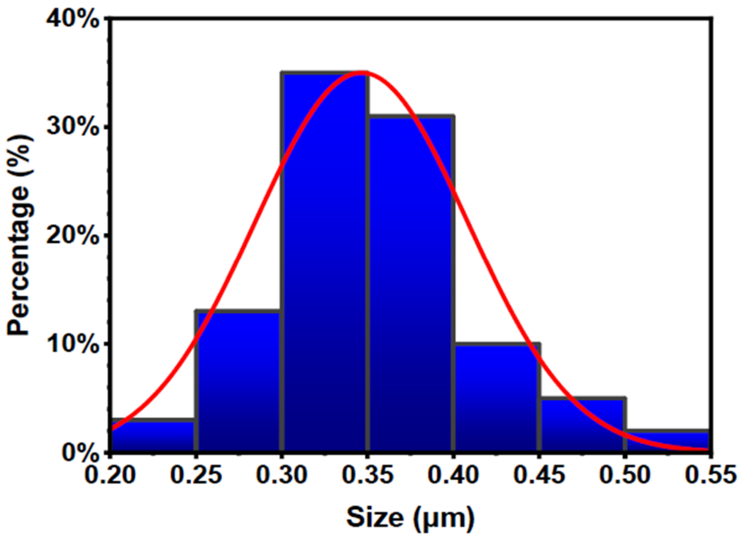

Figure S4. Gaussian distribution of the diameter of MFPA NPs.

6. The residual hemocytes on the MFPA NPs-coated cellulose acetate membrane.

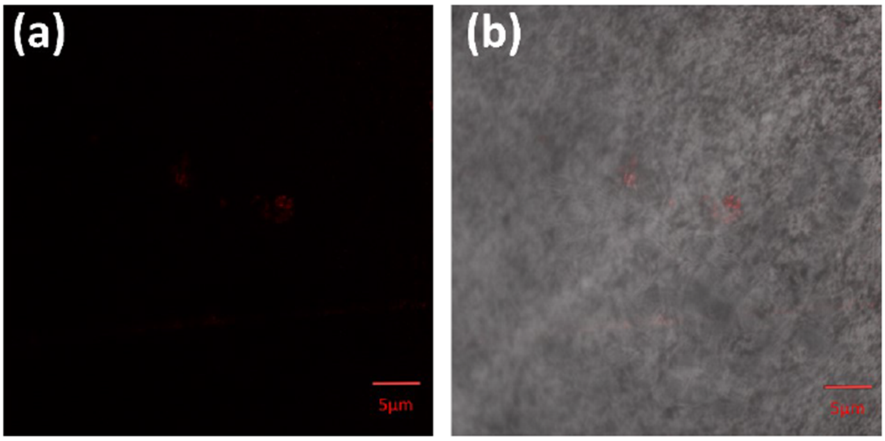

Figure S5. The laser confocal microscope images of MFPA NPs-coated cellulose acetate membrane after removing hemocytes by washing. (a) excited by 644 nm light, (b) bright field.

7. The AFM characterization of the cellulose acetate membrane coated by different amount of MFPA NPs

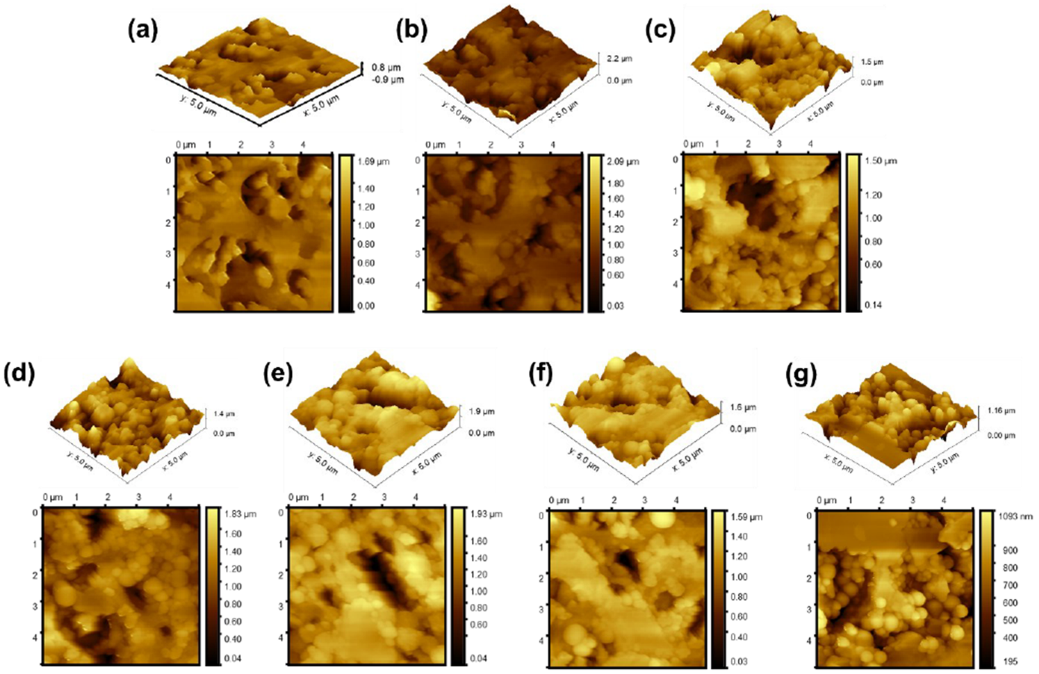

Figure S6. The AFM images of cellulose acetate membrane coated by different amounts of MFPA NPs (a) 0 mg/cm2, (b) 0.82 mg/cm2, (c) 1.17 mg/cm2, (d) 1.53 mg/cm2, (e) 1.88 mg/cm2, (f) 2.24 mg/cm2, (g) 2.59 mg/cm2.

8. The fluorescent microscope images of the cells adsorbed on the MFPA NPs-coated membrane.

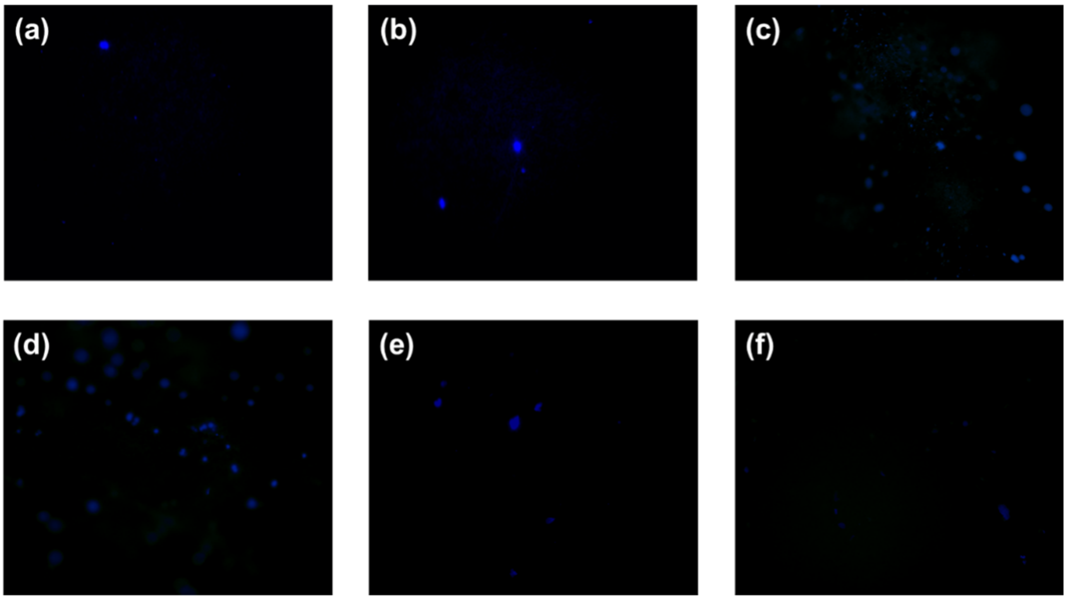

Figure S7. The fluorescent microscope images of the MCF-7 cells adsorbed on the MFPA NPs-coated membrane with different amounts of MFPA, (a) 0.82 mg/cm2, (b) 1.17 mg/cm2, (c) 1.53 mg/cm2, (d) 1.88 mg/cm2, (e) 2.24 mg/cm2, (f) 2.59 mg/cm2.

9. The oxTMB generated by different amount of MCF-7 cells

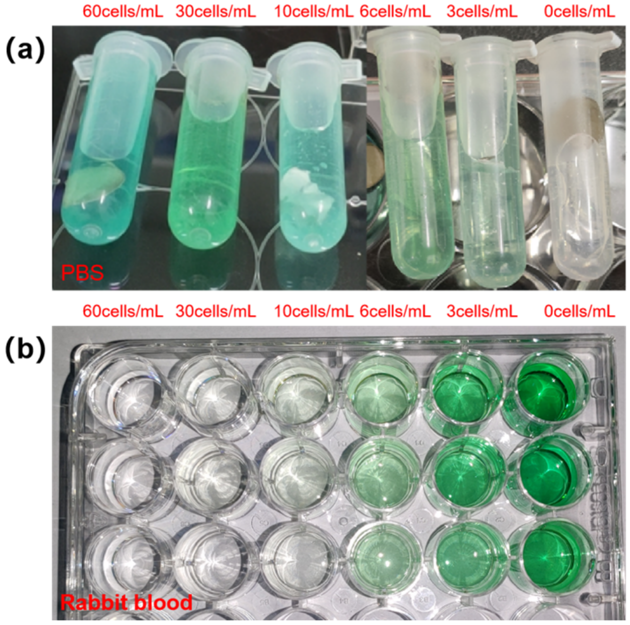

Figure S8. The enhancement of the UV-Vis absorbance with the increasing concentration of MCF-7 cells in PBS (a) or rabbit blood (b).
